# Genomic Characterization of Mobile Genetic Elements Associated With Carbapenem Resistance of *Acinetobacter baumannii* From India

**DOI:** 10.3389/fmicb.2022.869653

**Published:** 2022-06-15

**Authors:** Saranya Vijayakumar, Jobin John Jacob, Karthick Vasudevan, Purva Mathur, Pallab Ray, Ayyanraj Neeravi, Ashtawarthani Baskaran, Agilandeeswari Kirubananthan, Shalini Anandan, Indranil Biswas, Kamini Walia, Balaji Veeraraghavan

**Affiliations:** ^1^Christian Medical College & Hospital, Vellore, India; ^2^Jai Prakash Narayan Apex Trauma Center, All India Institute of Medical Sciences, New Delhi, India; ^3^Post Graduate Institute of Medical Education and Research (PGIMER), Chandigarh, India; ^4^Microbiology Department, Molecular Genetics and Immunology, University of Kansas, Lawrence, KS, United States; ^5^Indian Council of Medical Research (ICMR), New Delhi, National Capital Territory of Delhi, New Delhi, India

**Keywords:** CRAb, OXA–23, Tn*2006*, IC2, AbGRI1 variant, AbaR4

## Abstract

With the excessive genome plasticity, *Acinetobacter baumannii* can acquire and disseminate antimicrobial resistance (AMR) genes often associated with mobile genetic elements (MGEs). Analyzing the genetic environment of resistance genes often provides valuable information on the origin, emergence, evolution, and spread of resistance. Thus, we characterized the genomic features of some clinical isolates of carbapenem-resistant *A. baumannii* (CRAb) to understand the role of diverse MGEs and their genetic context responsible for disseminating carbapenem resistance genes. For this, 17 clinical isolates of *A. baumannii* obtained from multiple hospitals in India between 2018 and 2019 were analyzed. AMR determinants, the genetic context of resistance genes, and molecular epidemiology were studied using whole-genome sequencing. This study observed an increased prevalence of *bla*_*OXA–23*_ followed by dual carbapenemases, *bla*_*OXA–23*_, and *bla*_*NDM*_. This study identified three novel Oxford MLST sequence types. The majority of the isolates belonged to the dominant clone, IC2, followed by less prevalent clones such as IC7 and IC8. This study identified variations of AbaR4 and AbGRI belonging to the IC2 lineage. To the best of our knowledge, this is the first study that provides comprehensive profiling of resistance islands, their related MGEs, acquired AMR genes, and the distribution of clonal lineages of CRAb from India.

## Introduction

*Acinetobacter baumannii* is a member of the ESKAPE group of pathogens and is considered to be one of the major global causes of hospital-acquired infections (HAIs) (Lee et al., 2017). *A. baumannii* is responsible for causing a wide range of infections, with pneumonia being the most commonly observed infection among critically ill patients (Dexter et al., 2015). This pathogen has a propensity to rapidly acquire antibiotic resistance genes and to develop resistance to multiple classes of antimicrobials (Lee et al., 2017). Carbapenems are one of the most commonly used antibiotics for the treatment of *Acinetobacter* infections. Carbapenem resistance in *A. baumannii* ranges between 70 and 85% in the Asia-Pacific region (O’Donnell et al., 2021). A study from SENTRY surveillance reported carbapenem resistance rates ranging from 55 to 90% in India (Gales et al., 2019). Both the Center for Disease Control (CDC) and the World Health Organization (WHO) categorized carbapenem-resistant *A. baumannii* (CRAb) under “Urgent Threat” and as Priority 1: Critical pathogen, respectively. Recently, the WHO Country Office for India developed the Indian Priority Pathogen List (IPPL) and categorized carbapenem-resistant, colistin-resistant *A. baumannii* under “Critical Priority.”^1^

With excessive genome plasticity, *A. baumannii* can acquire and disseminate antimicrobial resistance (AMR) genes that are often associated with various mobile genetic elements (MGEs) (Roca et al., 2012). Carbapenem resistance in *A. baumannii* is mainly due to genes encoding class D oxacillinases, *bla*_*OXA–23–like*_, *bla*_*OXA–51–like*_, and *bla*_*OXA–58–like*_ (Poirel and Nordmann, 2006). The *bla*_*OXA–23*_ gene is the most predominant and is carried on many MGEs, including transposons, plasmids, and resistance islands (RIs) (Pagano et al., 2016). The association of insertion sequence (IS) elements with *bla*_*OXA–51*_-_*like*_, *bla*_*OXA–23–like*_, *bla*_*NDM–like*_, and *bla*_*OXA–58–like*_ genes was reported earlier (Poirel et al., 2005, 2012; Turton et al., 2006a). Typically, *bla*_*OXA*–23_ is associated with transposons such as Tn*2006*, Tn*2008*, and Tn*2009*, while *bla*_*NDM*–1_ was mobilized by the Tn*125*-like composite transposon (Pagano et al., 2016). Recent studies have also indicated the role of conjugative plasmids as vehicles for disseminating resistance determinants such as *bla*_*OXA–23*_ in *A. baumannii* (Salto et al., 2018). Additionally and most importantly, the emergence of *A. baumannii* RIs carrying clusters of horizontally transferred genes is considered a significant contributor to the multidrug-resistant (MDR) phenotype of *A. baumannii* (Cameranesi et al., 2020). RIs in *A. baumannii* are made of transposons and are known to carry genes that confer resistance to multiple antibiotics and heavy metals (Hamidian and Hall, 2018). The AbaR3-type elements are confined to the International Clone 1 (IC1) and represented by ST1, ST19, ST20, and ST81. AbaR3 comprises a Tn*6019* backbone and is consistently linked with Tn*6018* or its components with multiple antimicrobial resistance regions (MARRs) (Hamidian and Hall, 2011). Similarly, studies have shown that Tn*6022* can acquire *bla*_*OXA–23*_ transposon Tn*2006* and form AbaR4 islands (Hamidian and Hall, 2017). Table 1 outlines the genomic and epidemiological features of different clones of CRAb.

**TABLE 1:** Genomic and epidemiological features of different clones of carbapenem-resistant *Acinetobacter baumannii*–Indian vs. global scenario.

International clone (IC)	IC1		IC2		IC7		IC8	
	Indian	Global	Indian	Global	Indian	Global	Indian	Global
**Antimicrobial resistance (AMR) genes**	*bla*_*OXA–23*_, *bla*_*NDM–1*_	*bla*_*OXA–23*_, *bla*_*OXA–58*_	*bla*_*OXA–23*_, *bla*_*OXA–58*_, *bla*_*NDM–1*_	*bla*_*OXA–23*_, *bla*_*OXA–24*_, *bla*_*OXA–58*_, *bla*_*NDM–1*_	*bla*_*OXA–23*_	*bla*_*OXA–23*_, *bla*_*NDM–1*_	*bla*_*OXA–23*_	*bla*_*OXA–23*_, *bla*_*OXA–58*_, *bla*_*NDM–1*_
**Insertion sequence ISAba1**								
**Transposon**	Tn*6022*, Tn*2006*, Tn*125*	Tn*6018*, Tn*6019*, Tn*6022*, Tn*6172* Tn*2006*	Tn*6022*, Tn*6172*, Tn*2006*, Tn*6706*, Tn*6708*, Tn*125*	Tn*6022*, Tn*6172*, Tn*2006*, Tn*2007*, Tn*2008*, Tn*125*	Tn*6022*, Tn*2006*	Tn*6022*, Tn*6172*, Tn*6183*	Tn*6022*, Tn*2006*	Tn*6022*, Tn*6172*
**Resistance Island**	AbaR4	AbaR1, AbaR3, AbaR4, AbaR5 AbaR6, AbaR7, AbaR8, AbaR21, AbaR23, AbaR24	AbaR4, Novel AbGRIs	AbaR4, AbaR26, AbaR27, AbGRI1, AbGRI2, AbGRI3	AbaR4	AbGRI2	AbaR4	NA
**Predominant STs (Oxford MLST)**	ST231	ST109, ST207, ST231, ST405, ST441, ST491, ST781, ST945, ST947	ST848, ST208, ST195, ST451, ST218, ST369, ST349, ST1052	ST92, ST848, ST208, ST195, ST451, ST218, ST369	ST229, ST691, ST993	ST229, ST691	ST447, ST391, ST1390	ST447
**Predominant STs (Pasteur MLST)**	ST1	ST1	ST2	ST1	ST25	ST25	ST10	ST10
**Level of spread**	Low	Medium	High	High	Low	Low and region-specific	Low	Low and region-specific

Although the endemic burden of CRAb is a significant public health problem within Indian hospitals, the lack of genomic information makes it difficult to track its persistence (Mancilla-Rojano et al., 2019). Studying the genetic environment of resistance genes often provides valuable information on the origin, emergence, evolution, and spread of resistance in bacterial populations (Hamidian and Nigro, 2019).

We aimed to characterize the prevalent genomic features of clinical isolates of CRAb in India. We also compared the structural configuration of RIs with the complete genetic information and observed structural variations within the genetic environment of resistance genes. We found that the backbone of MGEs and their associated AMR genes among this study isolates were similar to that of the global context.

## Materials and Methods

### Bacterial Isolates

A total of 17 clinical isolates of *A. baumannii* collected as a part of a surveillance study were used. Of the 17 isolates included in this study, 13 were from Christian Medical College (CMC), Vellore, three from All India Institute of Medical Sciences (AIIMS) Trauma Center, New Delhi, and one from Post Graduate Institute of Medical Education & Research (PGIMER), Chandigarh (Supplementary Figure 1). Among the isolates, ten isolates were from blood (B; *n* = 10), six from endotracheal aspirate (ETA; *n* = 6), and one from pus (P; *n* = 1). Phenotypic characterization of all the isolates as *A. baumannii calcoaceticus* (*Acb*) complex was determined using standard biochemical tests. Confirmation of the *Acb* complex at the species level was performed by Vitek-MS (Database v2.0, bioMerieux, France) as described earlier and by identifying chromosomally encoded *bla*_*OXA–51*–*like*_ gene by PCR (Turton et al., 2006b).

### Antimicrobial Susceptibility Testing

All the isolates were characterized for susceptibility to ceftazidime (30 μg), cefepime (30 μg), piperacillin-tazobactam (100/10 μg), cefoperazone-sulbactam (75/30 μg), imipenem (10 μg), meropenem (10 μg), levofloxacin (5 μg), amikacin (30 μg), netilmicin (30 μg), tobramycin (10 μg), aztreonam (30 μg), tetracycline (30 μg), minocycline (30 μg), and tigecycline (15 μg) using the Kirby Bauer’s disk diffusion (DD) method.

For colistin, broth microdilution (BMD) was performed. Isolates identified as carbapenem-resistant by DD were further subjected to BMD to determine the minimum inhibitory concentration (MIC) for imipenem and meropenem. The susceptibility was interpreted as per the criteria defined by CLSI guidelines (Weinstein et al., 2018, 2019). *Escherichia coli* (ATCC 25922) and *Pseudomonas aeruginosa* (ATCC 27853) were included in every batch for quality control (QC). For colistin susceptibility testing, in addition to QC strains, an *mcr-1* positive *E. coli* isolate and two *Klebsiella pneumoniae* strains (BA38416 and BA25425) were also included for QC.

### Whole-Genome Sequencing and Assembly

The isolates were recovered overnight on blood agar, and genomic DNA was extracted from pure cultures using a QIAamp DNA mini kit (Qiagen, Germany) following the manufacturers’ instructions. DNA was quantified by NanoDrop spectrophotometry (Thermo Fisher Scientific, United States) and Qubit 3.0 fluorometry (Life Technologies, United States) and stored at −20°C until further characterization.

Short read sequencing of the 17 isolates was carried out using the Ion Torrent PGM™ platform using 400-bp chemistry (Life Technologies, United States) or 150-bp paired-end sequencing using HiSeq 2500 platform (Illumina, United States). The PHRED quality score was checked on the sequences, and reads with a score below 20 were discarded. Adapters were trimmed using cutadapt v. 1.8.1^2^ and assessed with FastQC version 0.11.4.^3^

All genomic DNA was further subjected to Oxford Nanopore MinION sequencing (Oxford Nanopore Technologies, United Kingdom) to obtain long-read sequences. For this, the DNA library was prepared as per the manufacturer’s protocol using the SQK-LSK108 ligation sequencing kit (version R9) and the ONT EXP-NBD103 Native Barcode Expansion Kit (Oxford Nanopore Technologies, United Kingdom). The library was loaded onto the FLO-MIN106 R9 flow cell and run for 48 h using the standard MinKNOW software. The Fast5 files from MinION sequencing were subjected to base calling using Guppy.^4^

Complete circular genomes for the 17 isolates were obtained using the *de novo* hybrid assembly of Illumina and Oxford nanopore sequences as described earlier (Wick et al., 2017a). The long reads were error-corrected using the standalone Canu tool (version 1.7) and filtered using Filtlong version 0.2.0^5^ with parameters set at *min_length 1000* −90 %. The short reads generated using ion torrent were error-corrected using Ionhammer (Ershov et al., 2019) available in SPAdes, and the default FastA output was converted to FastQ with custom scripts. Additionally, genomes were assembled using the Unicycler hybrid assembly pipeline (version 0.4.6) with the default settings (Wick et al., 2017b). The obtained genome sequence was polished using high-quality Illumina/Ion torrent reads to reduce the base level errors with multiple rounds of Pilon (version 1.22) (Walker et al., 2014). The assembly quality was assessed for completeness, correctness, and contiguity using CheckM version 1.0.5 (Parks et al., 2015). The genome sequences of the chromosomes and plasmids have been deposited in GenBank under the accession numbers–AB01 (CP040080–CP040083), AB02 (CP035672–CP035675), AB03 (CP050388–CP050389), AB06 (CP040050–CP040052), AB10 (CP040053–CP040055), AB11 (CP040056–CP040057), AB13 (CP040087–CP040088), AB14 (CP040259–CP040262), AB15 (CP050385–CP050387), AB16 (CP050523–CP050525), AB18 (CP050390–CP050391), AB19 (CP050410–CP050411), AB20 (CP050412–CP050414), AB23 (CP050432–CP050435), AB26 (CP050401–CP050402), AB27 (CP050421–CP050423), and AB28 (CP050403–CP050409).

### Genome Analysis

Further downstream analysis of the 17 complete genome sequences was performed using tools available at the Center for Genomic Epidemiology (CGE).^6^ AMR genes were identified from the genome sequences using the BLASTn-based ABRicate (version 0.8.10) program^7^ to query the ResFinder database.^8^ The Capsular Polysaccharide loci (KL) and the Outer Core Lipooligosaccharide loci (OCL) types were identified using the Kaptive database.^9^

The presence of ISs was identified using ISFinder.^10^ Using BLAST analysis, the plasmid rep*Aci* types from the complete genomes were identified and characterized. The PHASTER server was used to determine the prophages.^11^ The prophage regions identified were analyzed for the presence of any AMR genes using the ABRicate (version 0.8.10) program(see text footnote 7). A BLAST similarity search was performed on the individual genomes to identify the *comM* region that flanks AbaR type genomic islands. Based on the known AbaR sequences collected from published literature, the precise boundary of AbaRs and the respective backbone were curated manually. The sequence types were identified with MLST Finder 2.0 using Oxford MLST and Pasteur MLST schemes.^12^

### Phylogenetic Analysis

The assembled genome sequences were mapped to the reference genome ATCC 17978 (CP012004) using the BWA MEM^13^ algorithm, and the variants were filtered with FreeBayes available in Snippy (Seemann, 2015). The core SNP genome alignment of all the genomes was generated with Snippy-core. The recombination regions within the core genome alignment were further filtered and removed using the Gubbins (version 2.4.1) algorithm (Croucher et al., 2015). The maximum likelihood (ML) phylogeny was constructed using FastTree version 2.1.8 (Price et al., 2010) using the GTR model with 100 bootstrap replicates. The phylogenetic tree was rooted in the reference genome and labeled using the Interactive Tree of Life software (iTOL v3) (Letunic and Bork, 2021).

## Results

### Varied Resistance Profile of *Acinetobacter baumannii* Strains With *bla*_*OXA*–51_ and *bla*_*OXA*–23_ Variants

All 17 isolates were phenotypically identified as *Acb*-complex and further reconfirmed as *A. baumannii* using Vitek-MS (Data not shown). Among the 17 isolates, AB01 was pan-susceptible (PSAB), AB23 was multidrug-resistant (MDRAB) but susceptible to carbapenem (CSAB), 12 isolates (AB10, AB11, AB13, AB14, AB15, AB16, AB18, AB19, AB20, AB26, AB27, and AB28) were CRAb, and the remaining three isolates (AB02, AB03, and AB06) were pan-drug resistant (PDRAB). Table 2 outlines the presence of resistance genes among the 17 isolates against different classes of antimicrobials. All the study isolates carried the intrinsic *bla*_*OXA–51–like*_ and *bla*_*ADC–25*–like_ genes. Whole-genome sequencing (WGS) identified seven different variants of *bla*_*OXA–51*_ (*bla*_*OXA–66*_, *bla*_*OXA–68*_, *bla*_*OXA–64*_, *bla*_*OXA–144*_, *bla*_*OXA–203*_, *bla*_*OXA–337*_, and *bla*_*OXA–371*_), a single variant of *bla*_*OXA–23*_ (*bla*_*OXA–169*_), and a single variant of *bla*_*NDM–like*_ gene (*bla*_*NDM–1*_). Among the 17 study isolates, ten carried *bla*_*OXA–23*–*like*_ alone, one isolate carried *bla*_*NDM–1*_ alone, and four isolates co-harbored *bla*_*OXA–23*–*like*_ and *bla*_*NDM–1*_. No acquired carbapenemase genes were identified in the remaining two isolates. More than one copy of *bla*_*OXA–23*_ was observed in thirteen isolates (Table 2). As expected, none of our isolates carried *bla*_*OXA–24*–like_ or *bla*_*OXA–58*–like_ genes. The *A. baumannii* isolates in this study belonged to diverse sequence types (STs) representing four International clones, ICs (IC1/CC1, IC2/CC2, IC7/CC25, and IC8/CC10), one clonal complex, i.e., CC862, and one singleton (Table 2). Although genes that confer resistance to different antimicrobials were identified across all clonal lineages, the majority of the genes that encode for aminoglycoside modifying enzymes, RMTases, macrolide resistance, sulfonamide resistance, chloramphenicol, and rifampicin resistance were confined to isolates belonging to IC2 (Rodrigues et al., 2021; Hernández-González et al., 2022).

**TABLE 2:** Presence of antimicrobial resistance (AMR) genes, mobile genetic elements, and resistance islands among the 17 complete genomes of *Acinetobacter baumannii.*

Isolate ID (Accession number)	Specimen ID (ST Oxford/Pasteur)	Susceptibility	AMR gene profile	IS*Aba1*-*bla*_*OXA–51*_ like	*bla*_*ADC*_ allele*/IS*Aba1*-ADC	IS*Aba1*-*bla*_*OXA–23*_ like transposon	IS*Aba3*-*bla*_*OXA–58*_ like	IS*Aba125*-*bla*_*NDM–1*_ like transposon	Integron	Resistance Island (RI)
AB01 (CP040080)	SP304 (2439/285)	Pan-susceptible	*bla*_*OXA–337*_	–	*bla*_*ADC–33*_ (closest match)/Absent	–	–	–	–	Absent
AB02 (CP035672)	VB23193 (848/2)	PDR	*aac(6′)-Ib3*, *aadA1*, *aph(3″)-Ib*, *aph(6)-Id*, *armA*, *bla*_*OXA–169*_, *bla*_*OXA–23*_, *bla*_*OXA–66*_, *bla*_*PER–7*_ (2 copies), *mphE*, *msrE*, *catB8*, *cmlA1*, *aac(6′)-Ib-cr*, *ARR-2*, *sul1*, *sul2*, *tet(B)*	–	*bla*_*ADC–1*_ (closest match)/Present	Present–Tn*2006*	–	–	–	AbGRI-variant
AB03 (CP050388)	VB473 (848/2)	PDR	*aph(3″)-Ib*, *aph(3′)-Ia*, *aph(6)-Id*, *armA*, *bla*_*NDM–1*_, *bla*_*OXA–23*_ (2 copies), *bla*_*OXA–66*_, *mph(E)*, *msrE*, *sul2*, *tet(B)*	–	*bla*_*ADC–1*_ (closest match)/Present	Present–Tn*2006*	–	Present–Tn*125*	–	AbGRI1- variant
AB06 (CP040050)	VB16141 (2440/622)	PDR	*bla*_*NDM–1*_, *bla*_*OXA–203*_, *bla*_*OXA–23*_ (2 copies)	–	*bla*_*ADC–23*_ (closest match)/Absent	Present–Tn*2006*	–	Incomplete Tn*125*	–	AbaR4
AB10 (CP040053)	VB35179 (2392/586)	XDR	*bla*_*OXA–23*_ (2 copies), *bla*_*OXA–68*_	–	*bla*_*ADC–29*_ (closest match)/Present	Present–Tn*2006*	–	–	–	AbaR4
AB11 (CP040056)	VB35435 (2441/575)	XDR	*bla*_*OXA–144*_, *bla*_*OXA–23*_ (2 copies)	–	*bla*_*ADC–29*_ (Exact match)/Absent	Present–Tn*2006*	–	–	–	AbaR4
AB13 (CP040087)	VB35575 (349/2)	XDR	*aac(6′)-Ib3*, *aadA1*, *aph(3″)-Ib*, *aph(6)-Id*, *armA*, *bla*_*NDM–1*_, *bla*_*OXA–23*_ (2 copies), *bla*_*OXA–66*_, *mphE*, *msrE*, *catB8*, *cmlA1*, *aac(6′)-Ib-cr*, *ARR-2*, *sul1*, *sul2*, *tet(B)*	–	*bla*_*ADC–1*_ (closest match)/Present	Present–Tn*2006*	–	Present–Tn*125* like)	–	AbGRI- variant
AB14 (CP040259)	P7774 (1388/25)	XDR	*bla*_*OXA–23*_ (2 copies), *bla*_*OXA–64*_	–	*bla*_*ADC–23*_ (closest match)/Absent	Present–Tn*2006*	–	–	–	AbaR4
AB15 (CP050385)	VB82 (691/25)	XDR	*bla*_*OXA–23*_, *bla*_*OXA–64*_, *bla*_*TEM–1D*_, *mphE*, *msrE*, *tet(B)*	–	*bla*_*ADC–23*_ (closest match)/Absent	Present–Tn*2006*	–	–	–	AbaR4
AB16 (CP050523)	VB7036 (218/2)	XDR	*aph(3″)-Ib*, *aph(3′)-Ia*, *aph(6)-Id*, *armA*, *bla*_*NDM–1*_, *bla*_*OXA–23*_ (2 copies), *bla*_*OXA–66*_, *mphE*, *msrE*, *sul2*, *tet(B)*	–	*bla*_*ADC–1*_ (closest match)/Present	Present–Tn*2006*	–	Present–Tn*125* like	–	AbGRI1- variant
AB18 (CP050390)	VB723 (208/2)	XDR	*aph(3″)-Ib*, *armA*, *aph(3′)-Ia*, *aph(6′)-Id*, *bla*_*OXA–23*_ (2 copies), *bla*_*OXA–66*_, *bla*_*TEM–1D*_, *mphE*, *msrE*, *sul2*, *tet(B)*	–	*bla*_*ADC–1*_ (closest match)/Present	Present–Tn*2006*	–	–	–	AbGRI- variant
AB19 (CP050410)	PM2235 (451/2)	XDR	*aph(3″)-Ib*, *aph(3′)-Ia*, *bla*_*OXA–23*_ (2 copies), *bla*_*OXA–66*_, *bla*_*TEM–1D*_, *mphE*, *msrE*, *tet(B)*	–	*bla*_*ADC–1*_ (closest match)/Present	Present–Tn*2006*	–	–	–	AbGRI1- variant
AB20 (CP050412)	PM2696 (195/2)	XDR	*aph(3″)-Ib*, *aph(6′)-Id*, *bla*_*OXA–23*_ (2 copies), *bla*_*OXA–66*_, *mphE*, *msrE*, *sul2*, *tet(B)*	–	*bla*_*ADC–1*_ (closest match)/Present	Present–Tn*2006*	–	–	–	AbGRI- variant
AB23 (CP050432)	PM4229 (447/10)	MDR	*aph(3″)-Ib*, *aph(6)-Id*, *bla*_*OXA–68*_, *sul2*	–	*bla*_*ADC–29*_ (Exact match)/Absent	–	–	–	–	–
AB26 (CP050401)	VB2181 (195/2)	XDR	*aph(3″)-Ib*, *armA*, *aph(3′)-Ia*, *aph(6′)-Id*, *bla*_*OXA–23*_ (2 copies), *bla*_*OXA–66*_, *bla*_*TEM–1D*_, *mphE*, *msrE*, *tet(B)*	–	*bla*_*ADC–1*_ (closest match)/Present	Present–Tn*2006*	–	–	–	AbGRI1- variant
AB27 (CP050421)	VB2200 (451/2)	XDR	*aph(3″)-Ib*, *armA*, *aph(3′)-Ia*, *aph(6′)-Id*, *bla*_*OXA–23*_ (2 copies), *bla*_*OXA–66*_, *bla*_*TEM–1D*_, *mphE*, *msrE*, *tet(B)*	–	*bla*_*ADC–1*_ (closest match)/Present	Present–Tn*2006*	–	–	–	AbGRI- variant
AB28 (CP050403)	VB2486 (231/1)	XDR	*aph(3″)-Ib*, *aph(6′)-Id*, *bla*_*OXA–371*_, *bla*_*NDM–1*_, *sul2*	IS*Aba16* present but no insertional inactivation	*bla*_*ADC–27*_ (closest match)/Present	–	–	Present–Tn*125*	–	Tn*6022* derived elements

*PDR, pan drug-resistant; XDR, extensively drug-resistant; repAci, replicase type of Acinetobacter; IS, insertion sequence; Tn, transposon; tra, transfer genes; Mob, mobility genes.*

*The blaADC allele was identified using the ampC database incorporated in the PubMLST website: https://pubmlst.org/bigsdb?db=pubmlst_abaumannii_seqdef&page=sequenceQuery.

### IC2–the Predominant and Endemic Lineage With Novel Structural Variations

**FIGURE 1:**
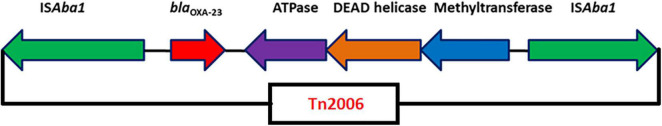
Genetic arrangement of *bla*_*OXA–23*_ identified in this study. The *bla*_*OXA–23*_ gene was flanked by two copies of an insertion sequence, IS*Aba1* in opposite orientations, forming a composite transposon, Tn*2006.*

**FIGURE 2:**
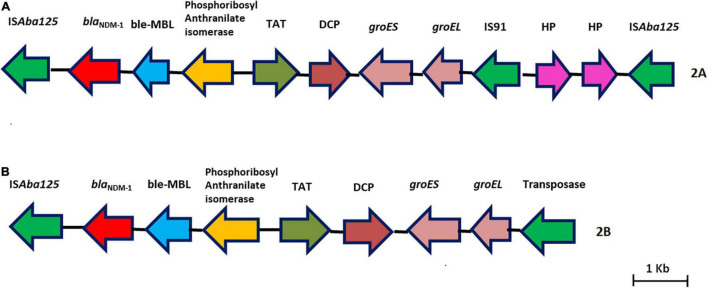
**(A,B)** Representative genomes showing the genetic environment of the *bla*_*NDM–1*_ gene characterized in this study. **(A)** Tn*125*–*bla*_*NDM–1*_ with two copies of IS*Aba125*. **(B)** Tn*125*-like–bla_*NDM–1*_ with one copy of IS*Aba125* and a truncated transposase.

Nine isolates belonged to IC2, and all had either XDR or PDR phenotypes. Six Oxford MLST STs (ST*^Oxf^*) were identified, ST195 (2), ST451 (2), ST848 (2), ST208 (1), ST218 (1), and ST349 (1), but there was a single Pasteur MLST ST2*^Pas^*. IC2 isolates predominantly carried either *bla*_*OXA–23*_ alone (7/9) or coproduced *bla*_*OXA–23*_ and *bla*_*NDM–1*_ (2/9). All had *bla*_*OXA*–23_ in Tn*2006*, an IS*Aba1*-bounded composite transposon in the chromosome (Figure 1). Of the nine isolates, AB03, AB13, and AB16 carried the *bla*_*NDM–1*_ gene on the chromosome with two different structural variations in the genetic context. AB03 and AB16 were associated with the most commonly reported transposon, Tn*125* (Figure 2A), while a truncated form of Tn*125* (Tn*125*-like) was identified in AB13, where the genome harbors a single copy of IS*Aba125* and an incomplete transposase at the left-hand and right-hand extremities of Tn*125*, respectively (Figure 2B). One to three plasmids were present among the nine genomes (Table 3). The p1AB20 belongs to the plasmid family which encodes *rep*Aci6 and was found to carry the *aphA6* gene within Tn*aphA6* that was bounded by two copies of IS*Aba125* in direct orientation. The most commonly observed prophage elements among the nine IC2 genomes include PHAGE_Acinet_Bphi_B1251_NC_019541, PHAGE_Psychr_Psymv2_NC_023734, and PHAGE_Acinet_ YMC11/11/R3177_NC_041866.

**TABLE 3:** Characteristic features of plasmids among the 17 complete genomes of *Acinetobacter baumannii.*

Isolate ID	Specimen ID (ST Oxford/Pasteur)	Susceptibility	Number of plasmids	Plasmid ID (Accession number)	*repAci* type	AMR gene profile	Virulence genes	Insertion sequence (IS) family	Integron	Others
AB01	SP304 (2439/285)	Pan-susceptible	3	p1AB01 (CP040081)	Frameshifted replication initiation protein	–	Sel1	–	–	–
				p2AB01 (CP040082)	Frameshifted replication initiation protein	–	Sel1	–	–	–
				p3AB01 (CP040083)	repAci3-97.89%	–	Sel1	–	–	–
AB02	VB23193 (848/2)	PDR	3	p1AB02 (CP035673)	RepM-Aci9	–	Septicolysin	–	–	–
				p2AB02 (CP035674)	RepM-Aci9-99.4%	–	Septicolysin	–	–	–
				p3AB02 (CP035675)	RepM-Aci9-99.79%	–	Septicolysin	–	–	–
AB03	VB473 (848/2)	PDR	1	p1AB03 (CP050389)	repAci4	–	Septicolysin	IS3	–	–
AB06	VB16141 (2440/622)	PDR	2	p1AB06 (CP040051)	Aci7-89.75%	aph(3″)-Ib, aph(6)-Id, armA, blaPER-7, mph€, msr€, cmlA1, ARR-2, sul1, sul2	–	IS4, IS91, IS6-like, IS5-like, Tn3-like	Class 1 (Int I1)	–
				p2AB06 (CP040052)	–	aph(3′)-VI				–
AB10	VB35179 (2392/586)	XDR	2	p1AB10 (CP040054)	–	aph(3″)-Ib, aph(6)-Id, armA, blaPER-7, mph€, msr€, cmlA1, ARR-2, sul1, sul2, tet(B)	–	IS5, IS6-like, Tn3-like, IS3, IS91-like, IS4-like	Class 1 (Int I1)	–
				p2AB10 (CP040055)	Aci4	–	Septicolysin	IS3	–	–
AB11	VB35435 (2441/575)	XDR	1	p1AB11 (CP040057)	–	aph(3′)-*via*, blaCARB-2, blaPER-7, sul1	–	IS6, IS91, IS30	–	–
AB13	VB35575 (349/2)	XDR	1	p1AB13 (CP040088)	repMAci9	–	Septicolysin	–	–	–
AB14	P7774 (1388/25)	XDR	3	p1AB14 (CP040260)	–	aac(6′)-Ian, aph(3″)-Ib, aph(6)-Id, armA, blaPER-7, mph€, msr€, cmlA1, ARR-2, sul1, sul2, tet(B)	–	IS5, IS4, IS70-like, IS91, IS6, IS30	Class 1 (Int I1)	–
				p2AB14 (CP040261)	A1S_3472	–	Septicolysin	IS3	–	–
				p3AB14 (CP040262)	–	–	–	–	–	–
AB15	VB82 (691/25)	XDR	2	p1AB15 (CP050386)	RepB family plasmid replication initiator protein (incomplete; partial on complete genome)	aac(6′)-Ian, aph(3″)-Ib, aph(6)-Id, armA, blaOXA-23, blaPER-7, mph€, msr€, cmlA1, ARR-2, sul1, sul2, tet(B)	MobL like, septicolysin	IS5, IS6, IS91, IS3, IS4, IS701-like	Class 1 (Int I1)	–
				p2AB15 (CP050387)	A1S_3472	–	–	–	–	–
AB16	VB7036 (218/2)	XDR	2	p1AB16 (CP050524)	RepA_AB	–	Sel1, Septicolysin			–
				p2AB16 (CP050525)	–	–	–			–
AB18	VB723 (208/2)	XDR	1	P1AB18 (CP050391)	AB57_3921	–	Sel1, Septicolysin			–
AB19	PM2235 (451/2)	XDR	1	P1AB19 (CP050411)	–	–	Sel1, Septicolysin			–
AB20	PM2696 (195/2)	XDR	2	p1AB20 (CP050413)	rep*Aci6*	aph(3′)-*via*	–	IS30 like		T4SS, type 4 *TraL*, *TraE*, *TraK*, *TraB*, *TraV*, *TraC*, *TraW*, *TraU*, *TrbC*, *TraN*, *TraF*, *TraH*, *TraG*
				p2AB20 (CP050414)	AB57_3921	–	Sel1, Septicolysin	–		–
AB23	PM4229 (447/10)	MDR	3	p1AB23 (CP050433)	–	aph(3″)-Ib, aph(6)-Id, armA, blaPER-7, mph€, msr€, cmlA1, ARR-2, sul1, sul2, tet(B)	MobL-like	IS4-like, IS5, IS91, IS26	Class 1 (Int I1)	Mercury operon, T6S protein, Conjugal transfer protein, TrbI, T4SS
				p2AB23 (CP050434)	Aci2-99.89%	–	MobA, MobL, Sel1, Septicolysin	–	–	–
				p3AB23 (CP050435)	repAci3	–	Sel1	–	–	–
AB26	VB2181 (195/2)	XDR	1	P1AB26 (CP050402)	AB57_3921	–	Sel1, Septicolysin			–
AB27	VB2200 (451/2)	XDR	2	p1AB27 (CP050422)	repAci1	–	Sel1, Septicolysin			–
				p2AB27 (CP050423)	–	aph(3′)-VI	MobA, mobL			–
AB28	VB2486 (231/1)	XDR	6	p1AB28 (CP050404)	plasmid replicase (rep*Aci6*) (PriCT_1” = “Primase C terminal 1 (PriCT-1)	blaOXA-23	IS21, IS256, IS66, IS4, IS30 like			T4SS, type 4 *TraL*, *TraE*, *TraK*, *TraB*, *TraV*, *TraC*, *TraW*, *TraU*, *TrbC*, *TraN*, *TraF*, *TraH*, *TraG* (Presence of repAci6 carrying AbaR4 with Tn2006-OXA-23)
				p2AB28 (CP050405)	repAci4	–	–			–
				p3AB28 (CP050406)	–	mph€, msr€	–			–
				p4AB28 (CP050407)	–	aph(3′)-VI	–			–
				p5AB28 (CP050408)	–	–	–			–
				p6AB28 (CP050409)	–	–	–			–

**FIGURE 3:**
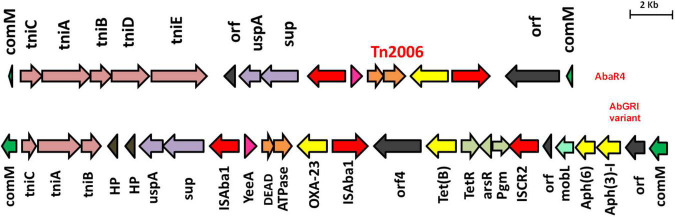
Structures of AbaR4 and variants of AbGRI1 identified in this study. The top figure depicts the typical AbaR4 island, while the bottom figure indicates the AbGRI1 variant due to additional *mobL* (light green arrow) and arsenic resistance gene, *arsR* (light gray arrow). The yellow arrow indicates antimicrobial resistance genes, and the red arrow depicts insertion elements.

**FIGURE 4:**
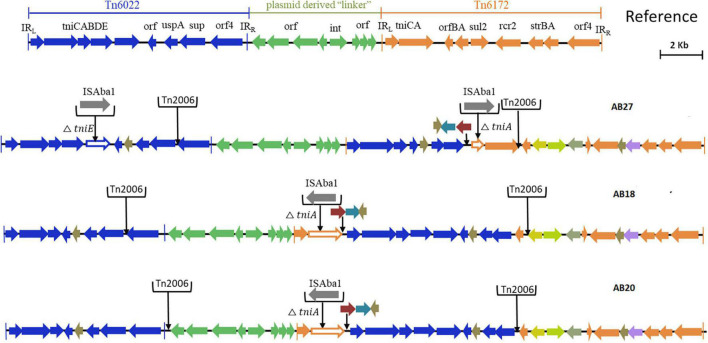
Structures of variants of AbGRI resistance island identified in this study. The typical AbGRI1 structure with an intact “Tn*6022*-linker-Tn*6172*” backbone is shown as a reference. The Tn*6022* or Tn*6022*-derived part is shown in blue, the Tn*6172* part or its partial segments are shown in orange, and the linker region is shown in green. The black arrows shown downward indicate the insertion of insertion sequence (IS) element or transposon or additional genes.

Based on the genetic configurations, three different variants of RIs were identified among the nine genomes. Of which, AB03, AB16, AB19, and AB26 carried variants of AbGR1, which included the presence of a partial region of Tn*6172* with the aminoglycoside resistance genes; *aph*(6) and *aph*(3)-I, mobilization gene; *mobL*, transposable element; CR2, phosphoglucosamine mutase; *pgm*, arsenic resistance encoding gene; *arsR*, tetracycline efflux protein; *tet(B)*, and tetracycline resistance transcriptional repressor gene; *tetR*(B) along with △Tn*6022* and Tn*2006* (Figure 3). AB18, AB20, and AB27 carried AbGRI variants with complex, diverse structures (Figure 4). AB18 and AB20 had a single copy of an IS*Aba1* element, *sul2*, *rcr2*, and a hypothetical protein inserted at the *tni*CA element on the Tn*6172* backbone. Two copies of Tn*2006* were observed in both the genomes but differed at the insertion site. In AB18, one copy of Tn*2006* was inserted at *orf4* while the second copy was inserted between *orf*BA and the tetracycline resistance transcriptional repressor gene, *tetR*(B). In AB20, one copy of Tn*2006* was observed between the Tn*6022* element and the plasmid linker, whereas another Tn*2006* was inserted near *orf*BA. AB27 had two copies of IS*Aba1*, one copy inserted at the *tniE* on the Tn*6022* backbone and the second copy inserted at *tniA* of Tn*6172*. Two copies of Tn*2006* were seen, one present on Tn*6022* at *orf4* with the second adjacent to *orf*BA on Tn*6172*. Insertion of *sul2*, *rcr2*, and hypothetical protein at the left inverted repeat of the Tn*6172* element was also observed. Additionally, insertion of the arsenic resistance encoding gene, *arsR*, and the mobilization gene, *mobL*, was present on the Tn*6172* element of all three genomes (Figure 4).

**FIGURE 5:**
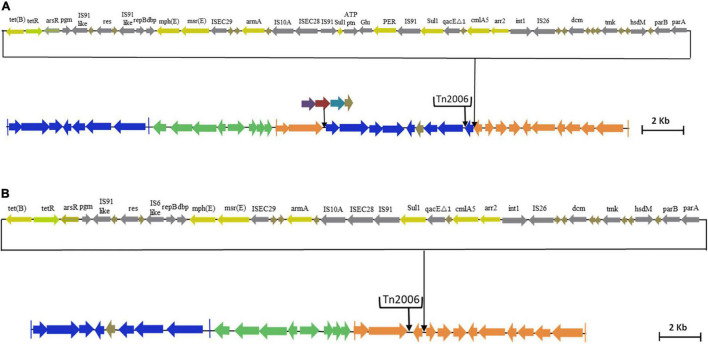
**(A)** Genetic backbone of AB13 carrying AbGRI variant identified in this study. The Tn*6022* or Tn*6022*-derived part is shown in blue, the Tn*6172* part or its partial segments are shown in orange, and the linker region is shown in green. The black arrows in the downward direction indicate the insertion of IS element, transposon, or additional genes. The pale yellow arrow indicates AMR genes, the light green arrow represents the tetracycline repressor gene, the gray arrow represents insertion elements, and the brown arrow indicates hypothetical protein. **(B)** Genetic backbone of AB02 carrying AbGRI variant identified in this study. The Tn*6022* or Tn*6022*-derived part is shown in blue, the Tn*6172* part or its partial segments are shown in orange, and the linker region is shown in green. The black arrows shown downward indicate the insertion of IS element or transposon or additional genes. The pale yellow arrow indicates AMR genes, the light green arrow represents the tetracycline repressor gene, the gray arrow represents insertion elements, and the brown arrow indicates hypothetical protein.

Interestingly, AB02 and AB13 carried the novel Tn*6022*-derived, plasmid linker, and Tn*6172*-derived elements. Insertion of a single copy of Tn*2006* and △CR2-△Tn10-MARR-like region in Tn*6172* was observed in both the genomes (Figures 5A,B). However, one minor difference was identified between the genomes, where AB02 carried *bla*_*PER–7*_ within the class 1 integron of the Tn*6172*-derived element (Figure 5A), while AB13 was found to carry a class 1 integron but was devoid of *bla*_*PER–7*_ (Figure 5B).

### IC7 and IC8–the Emerging Lineage of CRAb Isolates in India

Two genomes, namely, AB14 and AB15, belonged to IC7 and were represented by ST1388 *^Oxf^*/ST25*^Pas^* and ST691 *^Oxf^*/ST25*^Pas^*, and both were XDR. AB14 and AB15 carried three and two plasmids, respectively. AB14 harbored *bla*_*OXA–23*_ in Tn*2006* on the chromosome alone, whereas AB15 had it in both the chromosome (*bla*_*OXA–23*_ in Tn*2006*) and on an incomplete RepB family plasmid (*bla*_*OXA–23*_ in △Tn*2006*). Both AB14 and AB15 showed the presence of two prophage regions, namely, PHAGE_Mannhe_vB_MhM_3927AP2_NC_028766 and PHAGE_Acinet_YMC11/11/R3177_NC_041866. AbaR4 that were mapped to Tn*6022* backbone and Tn*2006* linked *bla*_*OXA–23*_ locus was present in both the genomes (Figure 3).

AB10, AB11, and AB23 belonged to IC8. Of the three, AB10 and AB11 were XDR and corresponded to novel STs: ST2392 *^Oxf^*/ST586*^Pas^* (AB10) and ST2441 *^Oxf^*/ST575*^Pas^* (AB11), whereas AB23 corresponded to ST447*^Oxf^*/ST10*^Pas^* and had an MDR phenotype. AB10 and AB11 were *bla*_*OXA–23*_ producers, while AB23 was found to be a non-carbapenemase producer. Two, one, and three plasmids were identified in AB10, AB11, and AB23, respectively. Three intact prophages such as PHAGE_Pelagi_HTVC010P_NC_020481, PHAGE_Acinet_Bphi_B1251_NC_019541, and PHAGE_Acinet_ vB_AbaS_TRS1_NC_031098 were seen in AB10. In AB11, PHAGE_Acinet_YMC11/11/R3177_NC_041866, PHAGE_ Acinet_Bphi_B1251_NC_019541, and PHAGE_Mannhe_ vB_MhM_3927AP2_NC_028766 were observed. A single prophage, PHAGE_Acinet_Bphi_B1251_NC_019541, was present in AB23. Similar to IC7, both XDR isolates carried AbaR4 on the chromosome but were absent in the MDR isolate, AB23 (Figure 3).

### IC1 Lineage With Tn6022-Derived Elements

**FIGURE 6:**
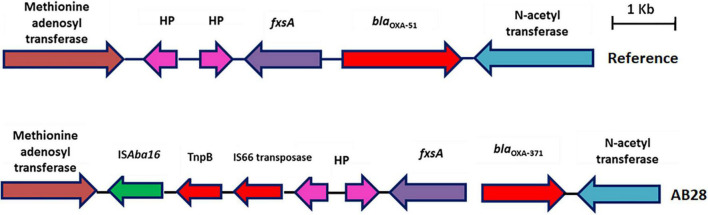
Genetic backbone of *bla*_*OXA–51*_. Two types of genetic structures were identified in this study. Sixteen isolates were identified with typical backbone, whereas one isolate with *bla*_*OXA–371*_ was identified with insertion sequence, IS*Aba16*, TnpB, and IS66 family transposase.

**FIGURE 7:**
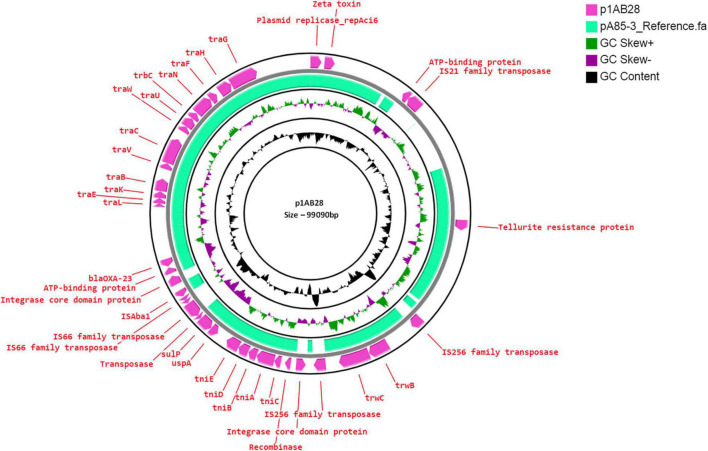
Circular representation of *rep*Aci6 plasmid (pink arrow), p1AB28, of *Acinetobacter baumannii* displayed using CG view server with the reference plasmid pA85-3 (accession number–KJ493819) (green-colored region). The two inner circles represent GC content and GC skew. The pink-colored arrow represents the presence of the OXA-23 gene along with the plasmid replication gene, repAci6, *tra* genes, and plasmid mobilization genes in p1AB28.

**FIGURE 8:**
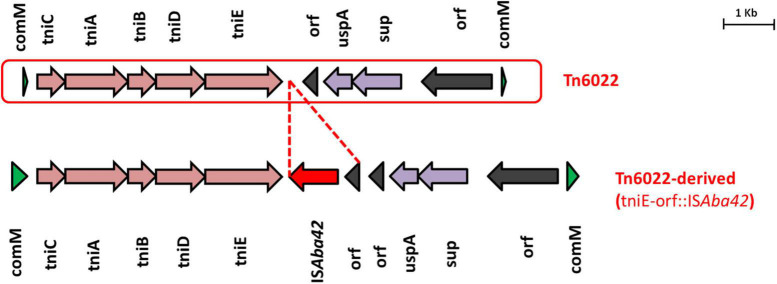
Structures of Tn*6022* and Tn*6022*-like elements. The typical Tn*6022* backbone is shown at the top of the figure. Tn*6022*-derived element observed in this study is displayed at the bottom of the figure and showed the insertion of IS*Aba42* (red arrow) with an additional *orf*. Appropriate names of the elements found within the genetic configurations are given. Dotted lines in red are used to depict the insertion of genetic elements.

AB28 had an XDR phenotype that corresponded to ST231 *^Oxf^*/ST1*^Pas^* and belonged to IC1. Interestingly, in AB28, which carried a variant of *bla*_*OXA–51*_ (*bla*_*OXA–371*_), an insertion of IS*Aba16*, TnpB, and an IS66 transposase, there was no upstream presence or insertional inactivation (Figure 6). AB28 carried Tn*125* linked *bla*_*NDM–1*_ on the chromosome (Figure 2A). AB28 harbored six plasmids. Of which, p1AB28 carried *bla*_*OXA–23*_ on *rep*Aci6 family plasmid and several plasmid transfer (*tra*) genes. When we analyzed and compared the p1AB28 plasmid sequence with the reference plasmid, pA85-3 (accession number–KJ493819), we found the presence of a complete *bla*_*OXA–23*_ gene with one complete and an incomplete copy of an IS*Aba1* locus. However, some transposon-related genes such as *uspA* and *sulP* were intact. IS66 family transposase with its accessory protein, *tnpB*, was also encoded within the p1AB28 plasmid but was absent in the pA85-3 reference plasmid. The plasmid, p1AB28, also carried putative *tra* genes that are required for mating pair formation and *trwC* and *trwB* genes that are needed for plasmid mobilization (Figure 7). AB28 carried five different prophages as follows: PHAGE_Stx2_c_Stx2a_F451_NC_049924, PHAGE_Acinet_Bphi_B1251_NC_019541, PHAGE_Psychr_ pOW20_A_NC_020841, PHAGE_Escher_SH2026Stx1_NC_ 049919, and PHAGE_Acinet_vB_AbaS_TRS1_NC_031098. Notably, AB28 encoded Tn*6022*-derived elements in which the insertion of an IS256 family transposase, IS*Aba42*, was observed between *tniE* and *orf* [Tn*6022* (*tniE-orf*)::IS*Aba42*] (Figure 8).

### Pan-Susceptible Singleton and Pan-Drug Resistant CC862

The PSAB, AB01 represented as a singleton and belonged to the novel ST, ST2439 *^Oxf^*/ST285*^Pas^*, while the PDRAB, AB06 belonged to CC862 and was represented by another novel ST, ST2440 *^Oxf^*/ST622*^Pas^*. As expected, AB01 did not harbor any of the AMR determinants except *bla*_*ADC–25*–like_, *bla*_*OXA–337*_. Three plasmids were present with no AMR genes. No intact prophage and RI were present.

AB06 carried dual carbapenemases, *bla*_*OXA–23*_ and *bla*_*NDM–1*_, on the chromosome, and they were found to be carried on transposon, Tn*2006*, and Tn*125*, respectively. Two plasmids were observed with genes encoding resistance to β-lactamases, aminoglycosides, macrolides, and sulfonamides. Three intact prophages, PHAGE_Pseudo_phiCTX_NC_003278, PHAGE_Acinet_Bphi_B1251_NC_019541, and PHAGE_Acinet_YMC11/11/R3177_NC_041866, were present. In addition, AB06 possesses the commonly reported AbaR4 RI (Figure 3).

### Phylogenetic Analysis of Core Genomes of CRAb

**FIGURE 9:**
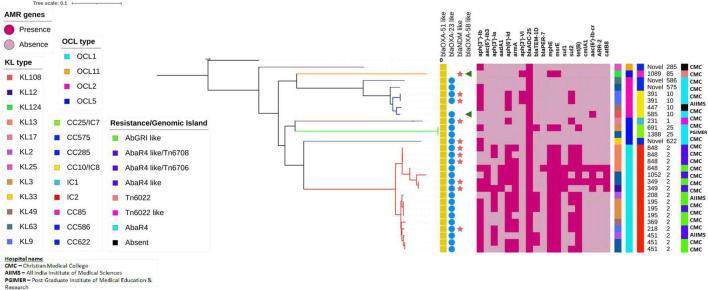
Single nucleotide polymorphism (SNP)-based phylogenetic tree of carbapenem-resistant *Acinetobacter baumannii* sequenced in this study. The color-filled shape denoted presence, while the empty shape denoted the absence of the respective traits. The heat map represents the presence or absence of AMR genes; Dark red indicates the presence of the respective gene, while light red indicates the lack of the respective gene. The capsular types (KL), outer core lipopolysaccharide types (OCL), International clones/clonal complexes, and resistance/genomic islands of the CRAb isolates were represented by color-coded boxes as given in the legend. The Oxford and Pasteur scheme sequence types (STs) were given as text labels.

Analysis of core genomes of CRAb revealed the presence of multiple AMR genes among the IC2 isolates. Clone-specific OCL types such as OCL1 to IC2, OCL5 to IC7, and OCL2 to IC8 were observed. Diverse KL types were identified among the study isolates, and the tree showed the presence of ST-specific KL types within a specific clonal lineage. AbaR4 was present among the IC1, IC7, and IC8 isolates, while AbaR4 and AbGRI variants were observed only among the IC2 isolates (Figure 9).

## Discussion

*Acinetobacter baumannii* has become an important hospital-acquired pathogen and is of major concern due to the rapid emergence of MDR, XDR, and PDR strains (Agoba et al., 2018; Havenga et al., 2019). Carbapenem resistance rates of more than 85% in *A. baumannii* have been reported from previous studies in India and are typically associated with isolates carrying either *bla*_*OXA–23*_ alone or both *bla*_*OXA–23*_ and *bla*_*NDM–1*_, which concurs with this study (Vijayakumar et al., 2016; Vijayakumar et al., 2019; Vijayakumar et al., 2020). The majority of the isolates (13/17) in this study encoded more than one copy of the *bla*_*OXA–23*_ gene. However, we could not find any high-level carbapenem resistance genes in these isolates. Earlier, Hua et al. (2016) reported the presence of multiple copies of *bla*_*OXA–23*_ among CRAb as a common phenomenon without an increase in carbapenem resistance.

This study showed the endemicity of IC2 along with the emergence of sporadic clones, such as IC7 and IC8. Although previous studies from India reported the predominance of IC2, the presence of isolates that belongs to IC7 and IC8 indicates the dissemination of CRAb and reinforces the fact that the International clones of CRAb isolates are widespread among hospitals in India.

Several studies have reported that the *bla*_*OXA–23*_ gene has relocated to chromosomes and plasmids with the help of transposons (Hamidian and Nigro, 2019; Graña-Miraglia et al., 2020). Fourteen CRAb isolates were identified with Tn*2006*-linked *bla*_*OXA–23*_ in this study. Although experimental observations were not performed, carbapenem resistance in these isolates could be due to the IS*Aba1*-mediated overexpression of the *bla*_*OXA–23*_ gene in Tn*2006*. Occasionally, carbapenem resistance in *A. baumannii* could happen due to the overexpression of *bla*_*OXA*–51_ variants by insertion of IS*Aba1* (Wong et al., 2019). In this study, the presence of IS*Aba16* was observed in one genome; however, insertional inactivation of *bla*_*OXA*–51–*like*_ was not seen.

In *A. baumannii*, *the bla*_*NDM–1*_ gene can be encoded by either chromosomes or plasmids (Bonnin et al., 2012). However, this study observed *A. baumannii* isolates harboring *bla*_*NDM–1*_ only in chromosomes. Unlike *Enterobacteriaceae*, in which *bla*_*NDM–1*_ is often observed with a single copy of truncated IS*Aba125* on plasmids, the dissemination of *bla*_*NDM–1*_ in *A. baumannii* is always associated with a complete Tn*125* (Poirel et al., 2012; Dortet et al., 2014). In contrast with the above statement, one genome in this study was identified with Tn*125*-_*like*_ linked *bla*_*NDM–1*_, suggesting that it could have acquired *bla*_*NDM–1*_ from other species.

The presence of *rep*Aci6 harboring *bla*_*OXA–23*_ and belonging to IC1 was identified in the p1AB28 plasmid. Comparative analysis revealed that AB28 carries a plasmid closely related to the reference, as it harbors *the bla*_*OXA–23*_ gene in a different context (Hamidian et al., 2016). The p1AB28 plasmid is conjugative and can spread carbapenem resistance by disseminating the *bla*_*OXA–23*_ gene into diverse clones. However, further studies are warranted to confirm the same. Another genome, AB20, belonged to IC2 and carried a *rep*Aci6 conjugative plasmid. This plasmid harbors the *aphA6* gene on the Tn*aphA6* transposon which encodes an aminoglycoside (3′) phosphotransferase and confers resistance to amikacin. Previous studies from European and Asian countries have reported isolates of *A. baumannii* with large conjugative plasmid such as rep*Aci6*, carrying both the *bla*_*OXA–23*_ and *aphA6* genes, which contribute to the dissemination of resistance to carbapenems and amikacin, respectively (Towner et al., 2011; Nigro and Hall, 2016). Earlier studies by Costa et al. (2018) reported the presence of AMR and virulence genes within the prophage regions of *A. baumannii* genomes. This study showed at least one prophage region in all the genomes except the PSAB. However, no prophages with AMR genes were detected.

Genomic analysis of AbaRs in this study unveiled novel genetic configurations specific to backbones, which involve either insertion of MGEs or structural modifications driven by known MGEs. For example, insertion of IS*Aba42* within the Tn*6022* backbone leads to a truncated form of the *tniE* transposition gene, thereby forming the Tn*6022* derived element. Furthermore, in this study, we identified an isolate (AB28) that belonged to IC1 but lacked an AbaR3 type island. Instead, it carried an IC2-specific Tn*6022*-derived backbone, which indicates the possibility of independent acquisition. Tn*6022*-derived elements and AbaR4 and AbGRI variants are typically confined to IC2. In this study, we also found that none of the IC2 isolates carried AbaR4; instead, it was present among isolates belonging to other ICs such as IC7 and IC8. All the study isolates belonging to IC2 possessed either the AbGRI1 variant or the AbGRI variant with complex chimeric structures. Although the genetic events behind this process are unclear, such complex structural variation in the AbaR backbones might have resulted either due to the target sequences favorable for MGE insertion or due to the exposure of AbaRs with different MGEs in different clones. These findings indicate that AbaRs with diverse backbones might have evolved separately.

## Conclusion

Overall, to the best of our knowledge, this study is the first that provides comprehensive profiling of RIs together with the MGEs, acquired AMR genes, and the distribution of clonal lineages among CRAb from India. Although this study provides a clear picture of the Indian scenario, further comparative analysis with an extensive collection of global isolates is required to understand the structural diversity and the evolution of these MGEs that drive the genome plasticity of *A. baumannii*.

## Data Availability Statement

The datasets presented in this study can be found in online repositories. The names of the repository/repositories and accession number(s) can be found in the article/Supplementary Material.

## Author Contributions

SV: laboratory methods, data analysis and interpretation, and manuscript writing. JJ: data analysis, interpretation, and manuscript writing. KV: hybrid genome assembly and other bioinformatics methods. PM, PR, SA, IB, and KW: manuscript correction. AN and AB: data analysis. BV: study design and supervising, manuscript writing, and manuscript correction. All authors contributed to the article and approved the submitted version.

## Conflict of Interest

The authors declare that the research was conducted in the absence of any commercial or financial relationships that could be construed as a potential conflict of interest.

## Publisher’s Note

All claims expressed in this article are solely those of the authors and do not necessarily represent those of their affiliated organizations, or those of the publisher, the editors and the reviewers. Any product that may be evaluated in this article, or claim that may be made by its manufacturer, is not guaranteed or endorsed by the publisher.

## Footnotes

1
https://dbtindia.gov.in

2
https://github.com/marcelm/cutadapt

3
http://www.bioinformatics.babraham.ac.uk/projects/fastqc

4
https://github.com/gnetsanet/ONT-GUPPY

5
https://github.com/rrwick/Filtlong

6
http://www.genomicepidemiology.org/services/

7
https://github.com/tseemann/abricate

8
https://cge.food.dtu.dk/services/ResFinder/

9
https://kaptive-web.erc.monash.edu/

10
https://www-is.biotoul.fr/blast.php

11
https://phaster.ca/

12
https://cge.food.dtu.dk/services/MLST/

13
https://github.com/lh3/bwa

## Supplementary Material

Supplementary Figure 1 A map of India showing the location of three hospitals from where the samples were collected as a part of the study. The red color represents PGIMER, Chandigarh, the green color represents AIIMS-Trauma, New Delhi, and the light red color represents CMC, Vellore. Map outline was created using mapchart.net. Republished from mapchart.net under a CC BY license, with permission from MapChart, original copyright 2021.
