# Supplementary figures and images for: Genomic Characterization of Mobile Genetic Elements Associated With Carbapenem Resistance of Acinetobacter baumannii From India
Source: Front Microbiol. 2022 Jun 15;13:869653. doi: 10.3389/fmicb.2022.869653 (PMC9240704; doi:10.3389/fmicb.2022.869653)

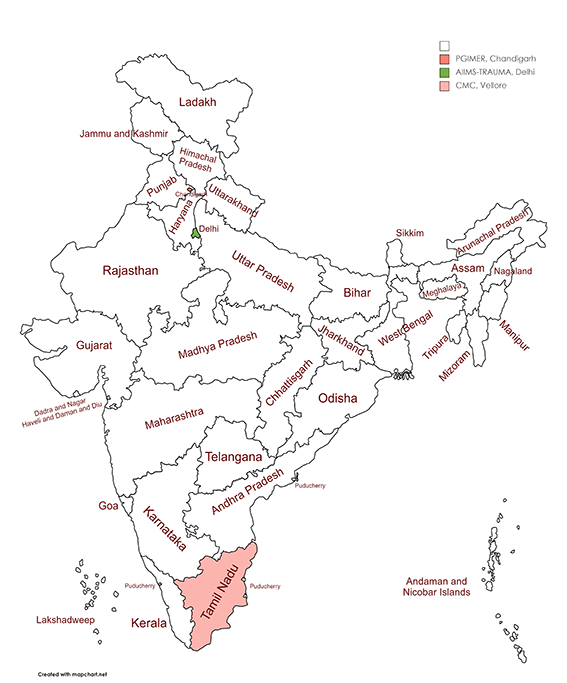

Supplement: Supplementary Figure 1 — A map of India showing the location of three hospitals from where the samples were collected as a part of the study. The red color represents PGIMER, Chandigarh, the green color represents AIIMS-Trauma, New Delhi, and the light red color represents CMC, Vellore. Map outline was created using mapchart.net. Republished from mapchart.net under a CC BY license, with permission from MapChart, original copyright 2021. [file Image_1.TIF]
